# Recycling Defunct Lithium‐Ion Battery Cathodes to Quaternary Layered Double Hydroxides for Efficient Oxygen Evolution Reaction

**DOI:** 10.1002/advs.202501957

**Published:** 2025-04-08

**Authors:** Ronghou Yao, Jin Wu, Shivam Kansara, Zhaowei Sun, Hyokyeong Kang, Feng Liu, Kaizhao Wang, Jin Hu, Xiangming Li, Dapeng Wu, Jang‐Yeon Hwang, Shizhao Xiong

**Affiliations:** ^1^ School of Environment Henan Normal University Xinxiang Henan 453007 China; ^2^ Faculty of Material Science and Engineering Kunming University of Science and Technology Kunming 650093 China; ^3^ Department of Energy Engineering Hanyang University Seoul 02581 South Korea; ^4^ Qujing No.1 Hospital Affiliated Qujing Hospital of Kunming Medical University No.1 Yuanlin Road Qujing Yunnan 655000 China

**Keywords:** cathodes recycle, electrocatalyst, layered double hydroxides, oxygen evolution reaction, spent LIBs

## Abstract

With the wide‐application of batteries (LIBs) correlated industries, continuous accumulation of wasted LIBs occurs, which causes environment issues and exhaustion of metal resources. Therefore, it is of vital importance to develop practical and efficient techniques to reuse the materials in wasted LIBs. In this work, a novel approach is proposed to reuse scrapped Li(Co_0.8_Ni_0.1_Mn_0.1_)O_2_ and LiFePO_4_ cathodes by converting them into oxygen evolution reaction (OER) electrocatalysts through simple leaching and co‐precipitation steps. The synthesized quaternary layered double hydroxides (Q‐LDHs) exhibits excellent OER activity, with the optimized Q‐LDH‐0.1 requiring a low overpotential of 270 mV to achieve the given current density of 10 mA cm^−2^, showing a low Tafel slope of 66 mV dec^−1^. The high catalytic activity of Q‐LDH‐0.1 should be attributed to the synergistic effects of electron interactions among homogeneously distributed iron, nickel, cobalt, and manganese atoms, the highly disordered coordination environment, and the catalytic activity of their mixed valence states, which combine to enhance the adsorption and transformation of OER intermediates. Additionally, the involvement of the more favorable lattice oxygen mechanism further promotes OER efficiency. This work presents a feasible approach for recycling abandoned LIB cathodic materials and offers valuable insights for developing low‐cost, highly‐effective LDH electrocatalysts.

## Graphical abstract

Li(Co_0.8_Ni_0.1_Mn_0.1_)O_2_ and LiFePO_4_ cathode materials are functional recycled by simple leaching and co‐precipitation methods to synthesize Quaternary iron‐nickel‐cobalt‐manganese hydrotalcites as oxygen‐evolution‐reaction catalysts. The highly disordered structure and strong electronic interactions between metal cations result in excellent catalytic performance. This research provides a feasible pathway for recycling end‐of‐life lithium battery cathodes and offers valuable scientific insight into oxygen‐evolution‐reaction electrocatalyst development.

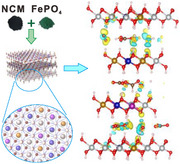

## 1 Introduction

With the rapid development of energy‐consuming societies, new‐energy batteries, particularly lithium‐ion batteries (LIBs), are increasingly applied to power electric vehicles, portable devices, and energy storage stations, making them one of the most essential parts of modern energy industries. Despite of the dominant role of LIBs in new‐energy industries, their overuse is giving rise to serious problems, such as environmental pollution and the exhaustions of valuable metal elements in electrode materials. For example, lithium (Li), nickel (Ni), cobalt (Co), and Manganese (Mn) are very commonly applied as the fundamental elements for commercial cathodes such as LiCoO_2_, LiMn_2_O_4_, and Li(Ni_1‐x‐y_Co_x_Mn_y_)O_2_.^[^ ^1^ ^]^ Therefore, improper disposal of the LIBs based on these cathodes will result in inevitable waste of valuable metal resources.

To make more effective utilization of metal elements in deserted cathodes, the most feasible approach is to develop effective recycling techniques, thereby repurposing them for new functions. The traditional recycling methods for defunct cathodic materials mainly include pyrometallurgy and hydrometallurgy.^[^ ^2^ ^]^ Pyrometallurgy is a well‐established technique widely adopted in industry for metal recovery through high‐temperature heating,^[^ ^3^ ^]^ yet is associated with issues like high energy‐consumption and poor consistency of final products.^[^ ^4^ ^]^ Hydrometallurgy typically begins with a leaching step to extract the metal elements using etchants, followed by post‐processing to obtain pure metal products. Therefore, such technique are complex and expensive.^[^ ^5^ ^]^ Given the disadvantages of the traditional methods, direct regeneration strategies have garnered significant attention in recent years, and a representative one among them is utilizing cathode leachates directly for the synthesis of electrocatalysts, for instance, oxygen evolution reaction (OER) catalysts.^[^ ^6^, ^7^, ^8^, ^9^ ^]^

The OER plays a crucial role in various clean energy technologies, such as fuel cells and metal‐air batteries. However, the OER involves a sluggish four‐electron transfer process, which requires significant overpotential to overcome the energy barrier.^[^ ^10^ ^]^ Therefore, highly efficient electrocatalysts are essential for OER as to reduce the reaction overpotential. Compared to conventional noble metal catalysts, transition metals and their compounds, have exhibited notable catalytic activity,^[^ ^11^, ^12^ ^]^ low cost and improved stability. Among these transition‐metal based materials, layered double hydroxides (LDHs), a type of anionic 2D material, have shown remarkable absorptivity/OER activity due to their unique layered structure and tunable metal ion composition.^[^ ^13^, ^14^ ^]^ The main structure layer of LDHs consists of divalent (M^2+^) and trivalent (M^3+^) metal cations in octahedral coordination with hydroxide ions, which can be replaced by other cations with similar valence states and ionic radii. Owing to such a tunable structure, multiple cations can be introduced into LDH structure, thereby producing plenty of defects and high‐energy sites and optimizing the catalytic activities. Especially, as revealed by recent works, the highly‐disordered LDHs containing at least four types of metal ions generally exhibit higher catalytic activity than conventional binary LDHs, owing to their diverse active sites, optimized electronic structure, and synergistic mechanism.^[^ ^14^, ^15^, ^16^ ^]^ Nevertheless, to fabricate a stable, homogenous, and highly‐disordered LDH is still a hard and costly task even for now. Based on all the discussions above, applying defunct cathode materials, which is rich in diverse transition metals, directly as precursors to produce highly disordered LDH‐based OER catalyst seems an ideal way to realize both resource recovery and renewable energy applications.^[^ ^17^, ^18^, ^19^ ^]^ However, research in this area is still lacking. Moreover, current strategies are generally complicated and costly. Therefore, Further studies are still necessary to develop more efficient and environment friendly recycling methods to reuse defunct cathode for synthesizing OER catalysts.

Herein, we propose a straightforward co‐precipitation method for synthesizing highly disordered quaternary hydrotalcite (Q‐LDHs) by applying deserted Li(Co_0.8_Ni_0.1_Mn_0.1_)O_2_ (NCM) and LiFePO_4_ (LFP) cathodes as raw materials. Specifically, a leachate containing Ni, Co, and Mn was obtained by the thermal leaching of NCM cathode with hydrochloric acid, which was then mixed with FePO_4_ obtained from scrapped LFP cathode to produce Q‐LDHs samples with varying iron (Fe) content. Owing to the similar ionic radii and coordination environment of Fe, Ni, Co, and Mn cations, all the Q‐LDHs samples exhibit relatively homogenous distribution of diverse metal elements, which combined to form catalysts with plenty of active sites and to realize the highly disordered structures as expected. Owing to the highly disordered structure and the synergetic interactions among diverse metal cations, all the Q‐LDH catalysts show good catalytic activity, suggesting the advantage of our strategy on recycling wasted cathodic materials and realizing high‐performance OER catalysts. Furthermore, by optimizing the content of Fe in the Q‐LDHs samples, the Q‐LDH‐0.1 sample is confirmed to involve the lattice oxygen mechanism (LOM) pathway during OER and exhibits exceptional catalytic activity, achieving an overpotential of 270 mV at a current density of 10 mA cm^−2^ in 1 m  potassium hydroxide (KOH) solution.

## 2 Results and Discussion

**Figure 1:**
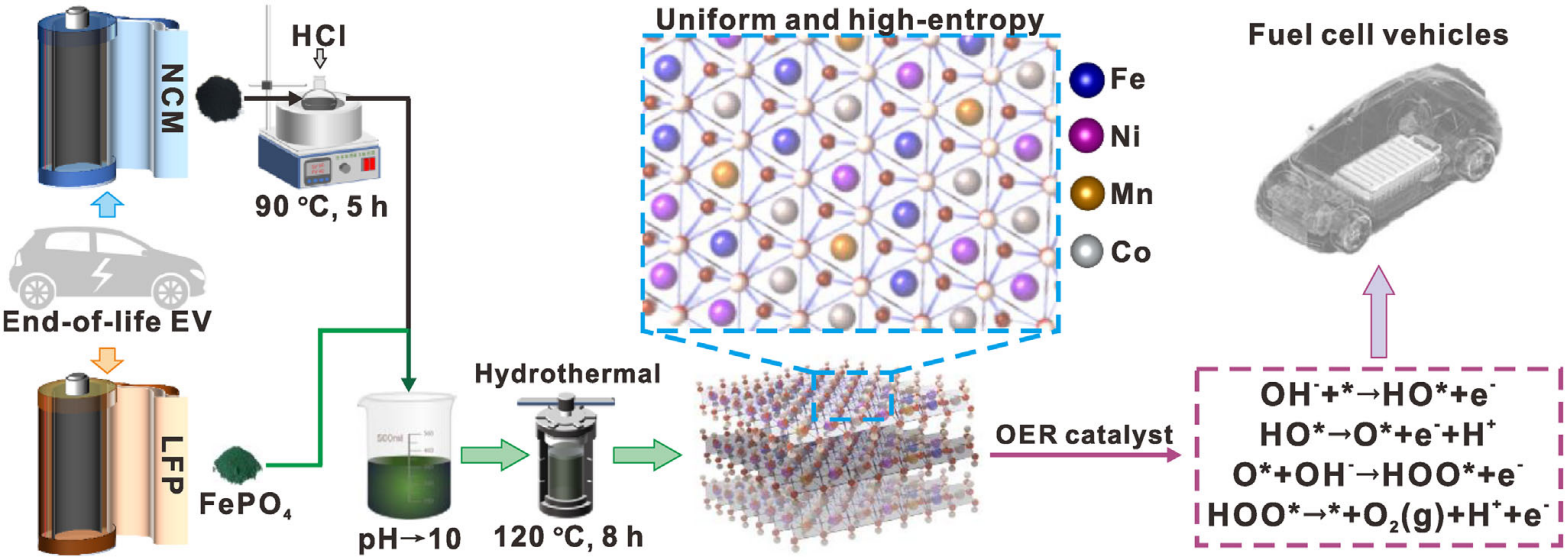
Schematic illustration of the general synthetic process of LDH catalysts from deserted cathodes of LIBs.

**Figure** **1** illustrates the synthesis process of ternary‐layered‐double‐hydroxide (T‐LDH) and Q‐LDHs samples, including the leaching process of the NCM cathode material and the following co‐precipitation and hydrothermal treatment. After the thermal leaching of NCM cathode with hydrochloric acid, Ni, Co, Mn, and Li ions remained in the leachate. Ni, Co, and Mn was precipitated in the subsequent co‐precipitation and hydrothermal processes, while Li remained in the solution and was separated (detailed procedures are available in the Experimental Section). We designated the sample without the addition of FePO_4_ during the synthesis process as T‐LDH, and the samples with varying amounts of FePO_4_ precursor were named as Q‐LDH‐0.01, Q‐LDH‐0.05, Q‐LDH‐0.1, Q‐LDH‐0.2, and Q‐LDH‐0.3, respectively.

**Figure 2:**
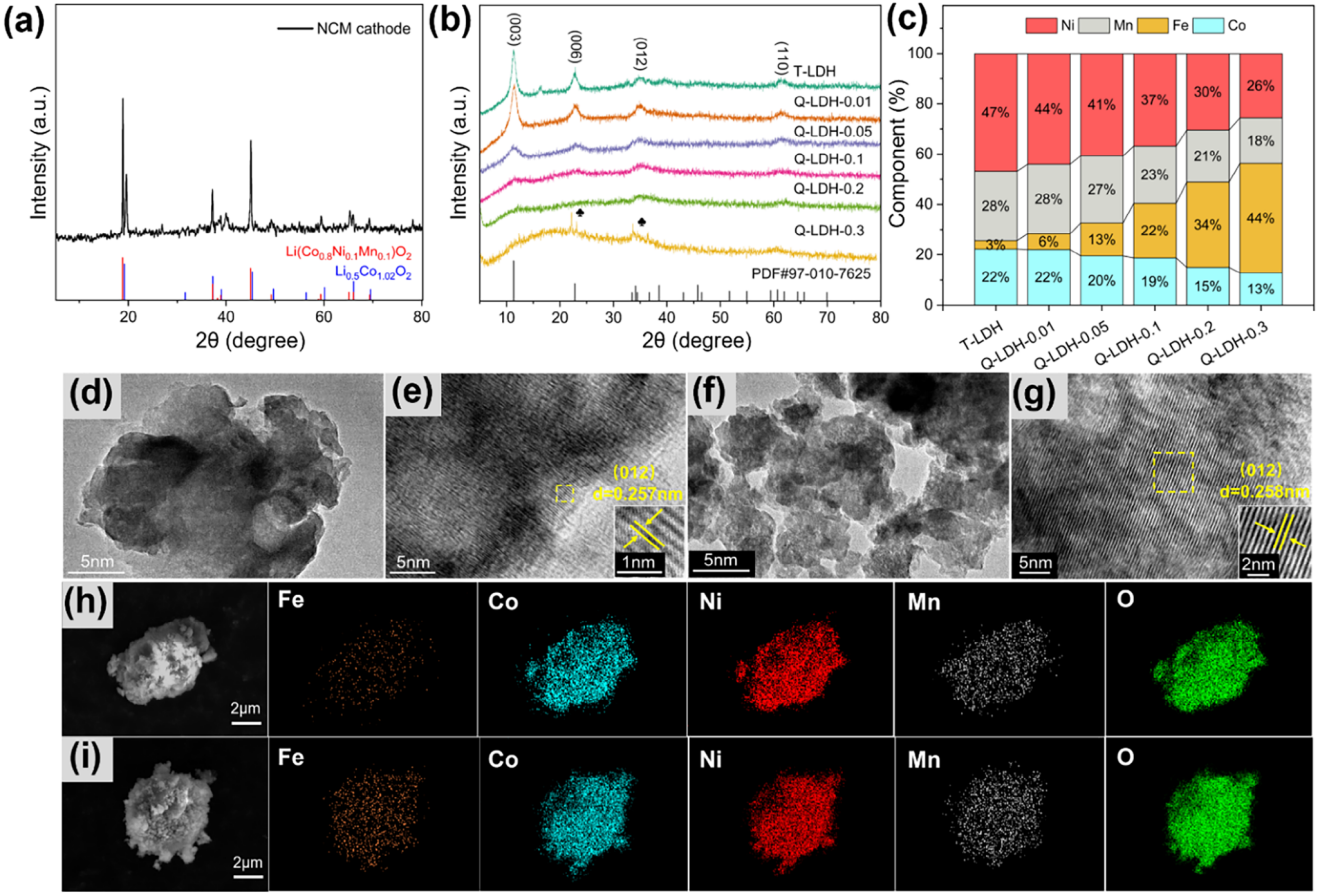
Structure and morphology characterization and element analysis of T‐LDH and Q‐LDHs. a) XRD pattern of the LIBs cathode. b) XRD pattern of the T‐LDH and Q‐LDHs. c) Proportion of metal components in T‐LDH and Q‐LDHs. d,e) HRTEM images of T‐LDH. f,g) HRTEM images of Q‐LDH‐0.1. h,i) Eds mapping images of T‐LDH and Q‐LDH‐0.1.

The X‐ray powder diffraction (XRD) pattern of NCM precursor (**Figure** **2**a) shows the coexistence of NCM and Li_0.5_Co_1.02_O_2_, while the latter may be previously applied as additive in NCM cathode. The XRD patterns of T‐LDH (Figure 2b) exhibit four distinct diffraction peaks at 11.5°, 23°, 35.1°, and 61.4°, corresponding to the (003), (006), (012), and (110) planes of LDHs, respectively. As previous studies demonstrated, such ordered structure adverse to realizing high catalytic activity compared to the disordered structure.^[^ ^20^, ^21^ ^]^ However, with the increasing content of Fe (inductively coupled plasma optical emission spectrometry (ICP‐OES) results in Figure 2c and Table S1, Supporting Information), the intensity of the characteristic peaks gradually decreases, indicating the decreased crystallinity and distortion of the ordered structure of Q‐LDHs.^[^ ^22^ ^]^ Such result is owing to the addition of the Fe(OH)_3_, which introduces diverse defects into the hydrotalcite structure, thus leading to the lattice distortion of the crystal structure and raising the internal energy and configuration/mixing entropy. In other words, the expected highly disordered structure is realized with the doping of Fe. Such result is certainly advantageous for improving the catalytic effect of Q‐LDH owing to the greatly increased active sites. Additionally, the small peak observed in the XRD pattern of Q‐LDH‐0.3 is likely caused by residual organic matter from the recovery process of the cathode materials.

Scanning electron microscopy (SEM) was used to observe the morphology of LDHs samples (Figure S1, Supporting Information). The images reveal that all the LDHs present block‐like appearance, which is likely due to the co‐precipitation of multiple metal elements at a time, leading to the agglomeration of Ni, Co, Mn, and Fe hydroxides during synthesis. As shown by the high‐resolution transmission electron microscopy (HRTEM) images (Figure 2d–g), T‐LDH and Q‐LDH‐0.1 both show clear lattice fringe of 0.257  and 0.258 nm, respectively, corresponding to the (012) plane of hydrotalcite, confirming the hydrotalcite crystal structure of both samples (Figure S2, Supporting Information). The element mappings of T‐LDH and Q‐LDH‐0.1 (Figure 2h,i) clearly show the existence of Fe, Co, Ni, and Mn in both samples, which are uniformly distributed throughout the surface. Such uniform dispersion of diverse metal elements proved the evenly mixing of the four metal components. Notably, the Fe observed in T‐LDH should be attributed to iron‐containing additives originally present in the NCM cathode that remained in the leachate after the leaching process.

**Figure 3:**
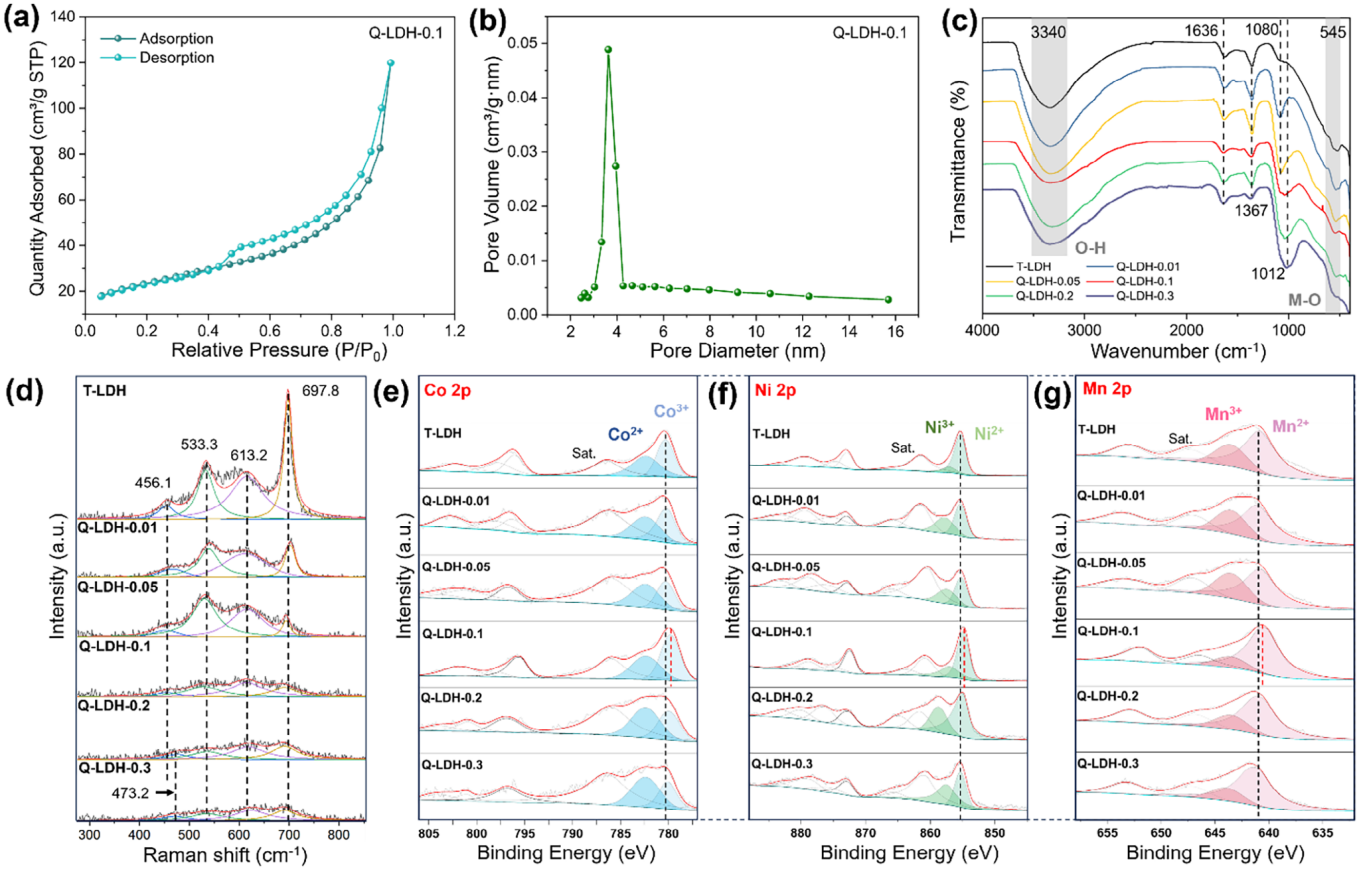
Structure and component analysis of T‐LDH and Q‐LDHs. a) Nitrogen adsorption–desorption isotherms. b) Pore‐size distributions. Chemical coordination and valency analysis of T‐LDH and Q‐LDHs. c) FT‐IR spectra. d) Raman spectra. e–g) XPS spectra of Co 2p, Ni 2p, and Mn 2p.

'Nitrogen adsorption–desorption isotherm analysis was performed to analyze the pore structure and adsorption character of Q‐LDH‐0.1, which exhibited Type IV isotherms characteristic of mesoporous materials, with a nitrogen adsorption–desorption isotherm analysis (BET) surface area of 83.1 m^2^ g⁻¹ (**Figure** **3**a). The large surface area is benefit for accelerating the adsorption process of OER reactants and optimize catalytic reaction. Figure 3b shows that the Q‐LDH‐0.1 depicted a narrow pore size distribution from 2 to 16 nm, ranging mainly 3.05–4.1 nm, further confirming the mesoporous nature of the material. Fourier transform infrared (FT‐IR) was used to analyze the chemical bond and functional group information of the catalyst samples, as shown in Figure 3c. The broad peak at 3340 cm^−1^ corresponds to the stretching vibration of O─H bonds of water molecules adsorbed between LDH layers, while the peak at 1636 cm^−1^ corresponds to the bending vibration of water molecules,^[^ ^23^, ^24^ ^]^ which is a typical phenomenon of common LDH materials. The peak at 1080 cm^−1^ is derived from the Cl^−^, SO_4_^2−^, and PO_4_^3−^ anions^[^ ^25^ ^]^ present in the raw materials. Additionally, a minor peak at 1367 cm^−1^ corresponds to the symmetric stretching of CO_3_^2−^,^[^ ^26^ ^]^ generated from CO_2_ adsorbed from the air during the synthesis process. The small peak at 545 cm^−1^ is related to the vibration of the metal‐oxygen bond (M─O).^[^ ^27^ ^]^ With the addition of Fe, the peak at 1080 cm^−1^ gradually intensifies, resulting in a more pronounced P‐O vibration of PO_4_^3−^. The peak shifts to a lower wavenumber when the concentration exceeds 0.1 m, likely due to the enhanced vibration of P‐OH (which occurs at a lower wavenumber than P‐O^[^ ^28^ ^]^), causing the P‐O and P‐OH vibrations to merge into a broader peak. Deducing from the FT‐IR results, although some impurities exist in the interlayer, the hydroxides layers are the dominate phases in the as‐prepared bulks.

Figure 3d shows the Raman spectra of T‐LDH and Q‐LDHs, which depict four characteristic peaks. The peaks at 456.1  and 533.3 cm^−1^ are attributed to the E_g_ bending mode of Ni–OH and A_1__g_ stretching mode of Ni–O in Ni(OH)_2_,^[^ ^29^ ^]^ respectively. The peaks observed at 613.2  and 697.8 cm^−1^ correspond to the Co‐O.^[^ ^18^, ^30^ ^]^ Notably, the peaks for Q‐LDH‐0.2 and Q‐LDH‐0.3 at 456.1 cm^−1^ shift to 473.2 cm^−1^, which can be attributed to the increased content of Ni(Fe)OOH.^[^ ^31^ ^]^ It is evident that as the Fe content increases, all peaks in the Raman spectrum gradually decrease and widen. This is likely due to the gradually weakening crystallinity of LDH, suggesting that the interaction between Fe(OH)_3_ and other metal–hydroxyl leads to the weakening of chemical bonding and the change of coordination environment. Such phenomenon is consistent with the XRD results, further proving the highly disordered structure realized by the doping of Fe, which would inspire the activity of diverse metal ions.

The elemental composition and chemical valence states of LDH are further investigated by X‐ray photoelectron spectroscopy (XPS). The survey spectra for all samples (Figure S3, Supporting Information) confirm the presence of Fe, Ni, Co and Mn in LDHs. As shown in Figure 3e, the Co 2p spectra exhibit two main peaks at 780.3  and 796.2 eV, attributed to the Co 2p_3/2_ and Co 2p_1/2_, respectively. Further, the Co 2p_3/2_ peak can be divided into two peaks at 780.3 eV (Co^3+^) and 782.4 eV (Co^2+^). The Co^2+^/Co^3+^ ratio (Peak area of Co^2+^ vs that of Co^3+^) is ≈1 for T‐LDH, which gradually decreases with increasing Fe content. Among all LDHs, Q‐LDH‐0.1 exhibits the lowest Co^2+^/Co^3+^ ratio of 0.68, with further increase of Fe content, the Co^2+^/Co^3+^ ratio gradually increases, likely due to the strong electronic interaction between Co and Fe. The Ni spectra (Figure 3f) show two peaks of Ni 2p_3/2_ at 857.9  and 855.2 eV, corresponding to Ni^3+^ and Ni^2+^, respectively, and one peak of Ni 2p_1/2_ at 873.1 eV. Compared with T‐LDH, the Ni^2+^/Ni^3+^ ratio in Q‐LDHs is smaller, indicating that the addition of Fe oxidizes more Ni^2+^ to Ni^3+^ in LDH, which primarily exists as NiOOH.^[^ ^32^ ^]^ Although previous studies have demonstrated that the OER catalytic activity of Ni^3+^ is higher than that of Ni^2+^,^[^ ^33^ ^]^ Ni^2+^ can be oxidized to Ni^3+^ during the OER process under applied voltage, thereby enhancing the catalytic activity of the catalyst. The Mn spectrum in Figure 3g shows the existence of both divalent and trivalent Mn, the peak at 641 eV is Mn 2p_3/2_, while the peak at 653 eV is Mn 2p_1/2_. Similar to Co and Ni spectra, the Mn 2p_3/2_ can be divided into two peaks at 643.5  and 641 eV, corresponding to Mn^3+^ and Mn^2+^, respectively. In T‐LDH, Q‐LDH‐0.01, and Q‐LDH‐0.05, the Mn^2+^/Mn^3+^ ratio remains approximately constant. With further increase in Fe content, the Mn^2+^/Mn^3+^ intensity ratios of Q‐LDH‐0.1, Q‐LDH‐0.2, and Q‐LDH‐0.3 exhibit a downward trend accompanied with the increased Fe^3+^, which occupies more trivalent cation sites and disturb the ordered crystal structure. The Fe 2p spectra (Figure S4, Supporting Information) indicates that Fe is mainly existed as Fe^3+^ in all samples. According to the XPS results, the metal cations in the LDHs show mixed valence state between bivalence and trivalence. Notably, in all the above‐mentioned spectra, the 2p_3/2_ peaks shift to relatively lower energy level with Q‐LDH‐0.1 (red dashed line), indicating the weaker chemical bonding between diverse metal cations and structural oxygen. Such phenomenon roots probably from the lattice distortion induced by Fe doping, which coincides with the XRD and Raman spectra.

**Figure 4:**
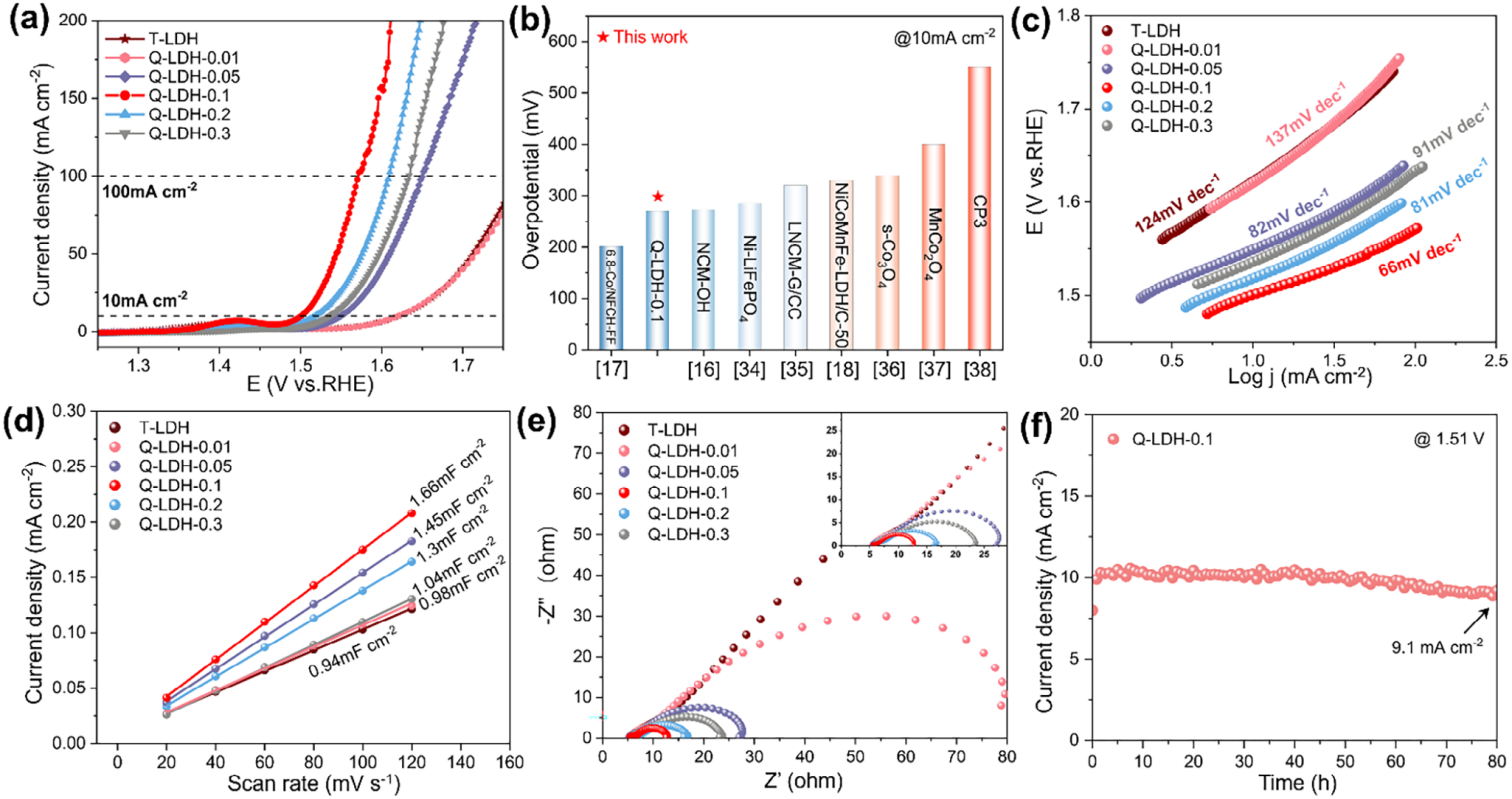
OER performance of T‐LDH and Q‐LDHs in 1 m KOH. a) LSV curves (the scan rate is 10 mV s^−1^) with 95% iR correction. b) Comparison of the catalytic activities of Q‐LDH‐0.1 with other OER catalysts synthesized from recovered LIBs cathodes as reported in previous studies. c) Corresponding Tafel slopes. d) Double‐layer capacitances (C_dl_). e) Electrochemical impedance spectrum. f) Stability test of Q‐LDH‐0.1 via chronoamperometry at 1.51V.

To evaluate the OER electrocatalytic performance of T‐LDH and Q‐LDHs, linear sweep voltammetry (LSV) curves were measured using a glassy carbon electrode coated with LDH as the working electrode in 1 m KOH solution (**Figure** **4**a). The detailed performance comparison is visible in Figure S5 (Supporting Information). Among all the LDH samples, Q‐LDH‐0.1 exhibits the best catalytic performance, requiring the smallest overpotential of 270 mV to achieve the given current density of 10 mA cm^−2^. This is significantly superior to T‐LDH (392 mV), Q‐LDH‐0.01 (391 mV), and Q‐LDH‐0.05 (320 mV), and even surpass the benchmark catalyst RuO_2_ (293 mV, Figure S6, Supporting Information). As the Fe content increases further, the catalytic performance begins to decline (Q‐LDH‐0.2, 288 mV and Q‐LDH‐0.3, 306 mV). Besides, Q‐LDH‐0.1 also outperforms all other LDHs at the higher current densities of 100 mA cm^−2^, which need a low overpotential of 340 mV, highlighting its potential as an excellent OER catalyst. The comparison of the performance between Q‐LDH‐0.1 catalyst in this study and catalysts reported in other studies that synthesized with recycled cathodes is shown in Figure 4b (The detailed comparison of electrochemical test conditions is presented in Table S2, Supporting Information), further demonstrating the superior and highly competitive performance of our work.^[^ ^16^, ^17^, ^18^, ^34^, ^35^, ^36^, ^37^, ^38^ ^]^ To further study the OER kinetics, Tafel slope curves were fitted based on the LSV data (Figure 4c). Q‐LDH‐0.1 exhibits the lowest Tafel slope of only 66 mV dec^−1^, which is significantly better than that of T‐LDH (124 mV dec⁻¹), Q‐LDH‐0.01 (137 mV dec⁻¹), Q‐LDH‐0.05 (82 mV dec⁻¹), Q‐LDH‐0.2 (81 mV dec⁻¹), and Q‐LDH‐0.3 (91 mV dec⁻¹). The low Tafel slope of Q‐LDH‐0.1 suggests it requires less voltage to achieve the prearranged current density, indicating its advantage on improving OER kinetics. Deducing from the worst performance of T‐LDH and Q‐LDH‐0.01, one can infer that the Fe sites function as the primary active sites for the catalyst, while the highly disordered Ni‐Co‐Mn hydroxides serve as the matrix,^[^ ^39^ ^]^ benefiting the catalytic performance enhancement. However, the relatively poor properties of Q‐LDH‐0.2 and Q‐LDH‐0.3 suggest that too extensively disordered hydroxide matrix will be somewhat harmful for the catalytic properties, which may result from the inhomogeneous structure and the declined electron transfer kinetics in highly disordered structure.

The electrochemical specific surface area (ECSA) can reflect the effective reaction area and the number of active sites in the catalyst. Cyclic voltammetry (CV) curves of T‐LDH and T‐LDHs were recorded at different scan rates within the voltage range of 0.1 to 0.2 V (Figure S7, Supporting Information). The double‐layer capacitance (C_dl_) values were obtained by fitting the CV curves. As shown in Figure 4d, Q‐LDH‐0.1 exhibits the highest C_dl_ value of 1.66 mF cm^−2^, compared to T‐LDH (0.94 mF cm^−2^), Q‐LDH‐0.01 (0.98 mF cm^−2^), Q‐LDH‐0.05 (1.45 mF cm^−2^), Q‐LDH‐0.2 (1.30 mF cm^−2^), and Q‐LDH‐0.3 (1.04 mF cm^−2^). These results suggest that Q‐LDH‐0.1 possesses the highest ECSA, which increases the exposure of active sites and facilitates the transport of OH^−^ and O_2_ during the OER process, thus contributing to the superior electrocatalytic activity of Q‐LDH‐0.1.

To further evaluate the electron transfer rate and reaction kinetics during the OER process, electrochemical impedance spectroscopy (EIS) was conducted on the catalysts (Figure 4e). As seen in the Nyquist plot, Q‐LDH‐0.1 has the smallest semicircular diameter, indicating the lowest charge transfer resistance, which corresponds to a faster catalytic rate. Electrocatalytic stability is a crucial factor for both performance evaluation and the potential of practical application. Q‐LDH‐0.1 exhibited satisfactory durability, retaining 91% of its initial current density after an 80‐h chronoamperometry test, as shown in Figure 4f. This result suggests its potential as a credible electrocatalyst for long‐term applications.

**Figure 5:**
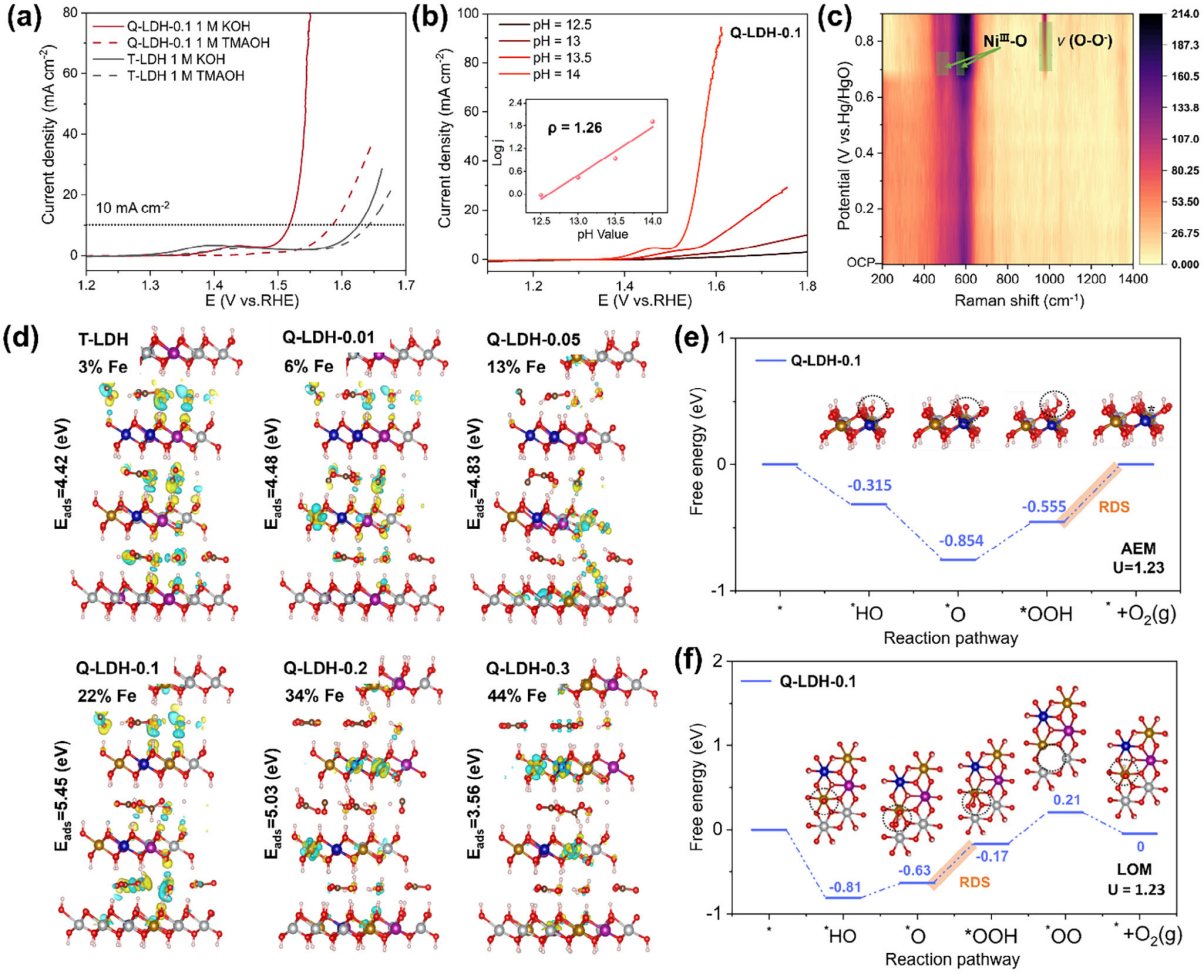
Experimental verification and theoretical calculations of OER reaction mechanism for T‐LDH and Q‐LDHs catalysts. a) LSV curves of Q‐LDH‐0.1 and T‐LDH recorded in 1 m KOH and 1 m TMAOH electrolytes, respectively. b) LSV curves of Q‐LDH‐0.1 were measured at different pH values (12.5‐14). The inset shows the fitted slope of the linear plot of the logarithm of current versus pH at 1.6 V, representing the proton reaction order (ρ). c) in situ Ramam spectra of Q‐LDH‐0.1. d) Charge density difference contour plots. The structure models of T‐LDH and Q‐LDHs were composed of NiFe_x_MnCo (x = 3%, 6%, 13%, 22%, 34%, and 44%, based on ICP‐OES results). Free energy diagrams of e) AEM and f) LOM pathways at U = 1.23 V on Q‐LDH‐0.1. In this calculation method, the free energy difference between two steps divided by *e* represents the theoretical overpotential for that transformation step. The step with the highest theoretical overpotential is identified as the rate‐determining step, as shown in the orange shaded area.

In general, the LDH catalysts follow the adsorbate evolution mechanism (AEM) during OER process, involving a series of adsorption and desorption steps of oxygenated intermediates (Figure S8a, Supporting Information). However, AEM is inherently constrained by the linear scaling relationship between intermediates and active site, which limits the catalytic efficiency.^[^ ^40^ ^]^ In contrast, LOM, a mechanism in which lattice oxygen directly participates in the OER reaction and forms O‐O coupling, could help circumvent such constraints, leading to significantly lower overpotentials (Figure S8b, Supporting Information). To gain deeper insight into the OER mechanism of our catalyst and elucidate the origin of its excellent OER activity, we conducted mechanistic studies through both experimental and simulation approaches. The formation of O_2_^2−^ species, a key feature of LOM, is known to exhibit strong electrostatic interactions with tetramethylammonium cations.^[^ ^41^ ^]^ Therefore, tetramethylammonium hydroxide (TMAOH) can serve as a diagnostic probe for LOM, as it binds O_2_^2−^ species and thereby inhibits OER kinetics. Based on this principle, we performed LSV measurements in TMAOH electrolyte and compared the results with those obtained in KOH, as shown in **Figure** **5**a. The overpotential of Q‐LDH‐0.1 increased by ≈70 mV at 10 mA cm^−2^ in TMAOH, whereas T‐LDH exhibited only a minor increase of 16 mV. This pronounced suppression of OER kinetics in Q‐LDH‐0.1 strongly suggests that it follows the LOM pathway. Furthermore, previous studies have demonstrated that LOM is pH‐dependent while AEM is pH‐independent,^[^ ^42^ ^]^ thus we performed pH‐dependent LSV measurements. As depicted in Figure 5b and Figure S9 (Supporting Information), Q‐LDH‐0.1 displayed a clear pH dependence, with a proton reaction order (ρ) of 1.26, significantly surpassing the T‐LDH (ρ = 0.39). This substantial difference further confirms that Q‐LDH‐0.1 follows the LOM pathway during OER. According to the in situ Raman spectra in Figure 5c and Figure S10 (Supporting Information), the characteristic peaks at ≈470 and 550 cm^−1^, attributed to Ni^III^‐O vibrations of NiOOH, exhibit a progressive increase in intensity with rising applied potential. This enhancement becomes particularly pronounced beyond 0.7 V (vs Hg/HgO), coinciding with the emergence of the γ‐CoOOH characteristic peak at 600 cm^−1^. Previous studies have elucidated that transition metal‐based catalysts would undergo such in situ metal oxyhydroxides evolution under electrochemical water oxidation conditions.^[^ ^43^ ^]^ Simultaneously, the band at ≈970 cm^−1^ intensifies markedly, signifying the formation of active oxygen species during the OER process, which originate from the deprotonation of oxyhydroxides.^[^ ^44^ ^]^ These spectral evolutions suggest that the metallic sites, particularly Ni within the LDH, undergo progressive oxidation to higher valence states under anodic conditions, thereby enhancing catalytic activity. This observation indicates the involvement of oxyhydroxides species during the OER process and the participation of AEM, as AEM is primarily driven by high‐valence metal active sites. Consequently, the catalytic process of Q‐LDH‐0.1 integrates both AEM and LOM, which endows Q‐LDH‐0.1 with markedly enhanced catalytic activity compared to T‐LDH.

To gain a deeper understanding of the impact of Fe doping and the synergistic effect of multi‐metallic sites on catalytic activity, theoretical calculation was performed. The structure models with different Fe content of 3%, 6%, 13%, 22%, 34%, and 44% correspond to T‐LDH, Q‐LDH‐0.01, Q‐LDH‐0.05, Q‐LDH‐0.1, Q‐LDH‐0.2 and Q‐LDH‐0.3, respectively (Figure S11, Supporting Information). Fe doping levels ranging from 3% to 44% induce notable modifications in the Density of States (DOS) near the Fermi level (0 eV), resulting in an elevated density of states in this region, especially for Q‐LDH‐0.1. This indicates improved electronic conductivity, which is critical for facilitating charge transfer during OER. The incorporation of Fe introduces new electronic states near the Fermi level and facilitates the hybridization of Fe 3d orbitals with Ni, Co, and Mn 3d orbitals, which forms active electronic centers that are essential for catalytic activity. Furthermore, spin‐polarized DOS reveals asymmetry between spin‐up and spin‐down components, suggesting potential spin polarization effects that could influence the catalytic mechanism. These electronic structure modifications suggest that Fe serves as a highly reactive site in AEM pathway, capable of lowering the energy barriers for key OER intermediates such as ^*^OH, ^*^O, and ^*^OOH. The results highlight the synergistic interaction between Fe and the host LDH framework, which collectively contributed to the enhanced OER catalytic performance of the material. Charge density difference (CDD) analysis was conducted to reveal the electronic and structural properties of T‐LDH and Q‐LDHs (Figure 5d). Catalytic performance is intrinsically related to the ability for adsorbing and activating key reactants such as H_2_O, as well as the electronic and charge transfer properties. The CDD contours show accumulation and depletion near interlayer anions, indicating an active redistribution of electronic density, which facilitates the adsorption and activation of H_2_O and reaction intermediates. Notably, the introduction of Fe significantly enhances the adsorption energy, reaching a maximum of −5.45 eV for Q‐LDH‐0.1. This improved adsorption indicates stronger interactions between the active sites and oxygenated intermediates, which makes the final step of AEM (oxygen generation and desorption) the rate‐determining step (RDS), with thermodynamic overpotential of 0.55 V (Figure 5e). Noteworthy, in the highest Fe‐doped system (44%), the charge density shifts predominantly to the metallic sites, leaving the adsorbed H_2_O electronically under‐activated. This suggests that excessive Fe doping could result in suboptimal charge transfer and reduced catalytic efficiency for OER. Suitable Fe doping not only modulates the electronic structure of the LDH, introducing additional states near the Fermi level that are crucial for facilitating electron transfer during the OER, but also triggers the LOM pathway. As shown in Figure 5f, in the LOM pathway, the RDS shifts to the ^*^OOH→^*^OO transition (≈0.46 V), which is lower than the overpotential of the RDS in the AEM pathway, making it thermodynamically more favorable. Consequently, Q‐LDH‐0.1, which incorporates the LOM pathway, exhibits significantly higher catalytic performance than T‐LDH. The enhancements in adsorption energy, charge redistribution, electronic structure, and the involvement of the LOM pathway in the optimal sample Q‐LDH‐0.1 work synergistically to elevate its catalytic performance.

**Figure 6:**
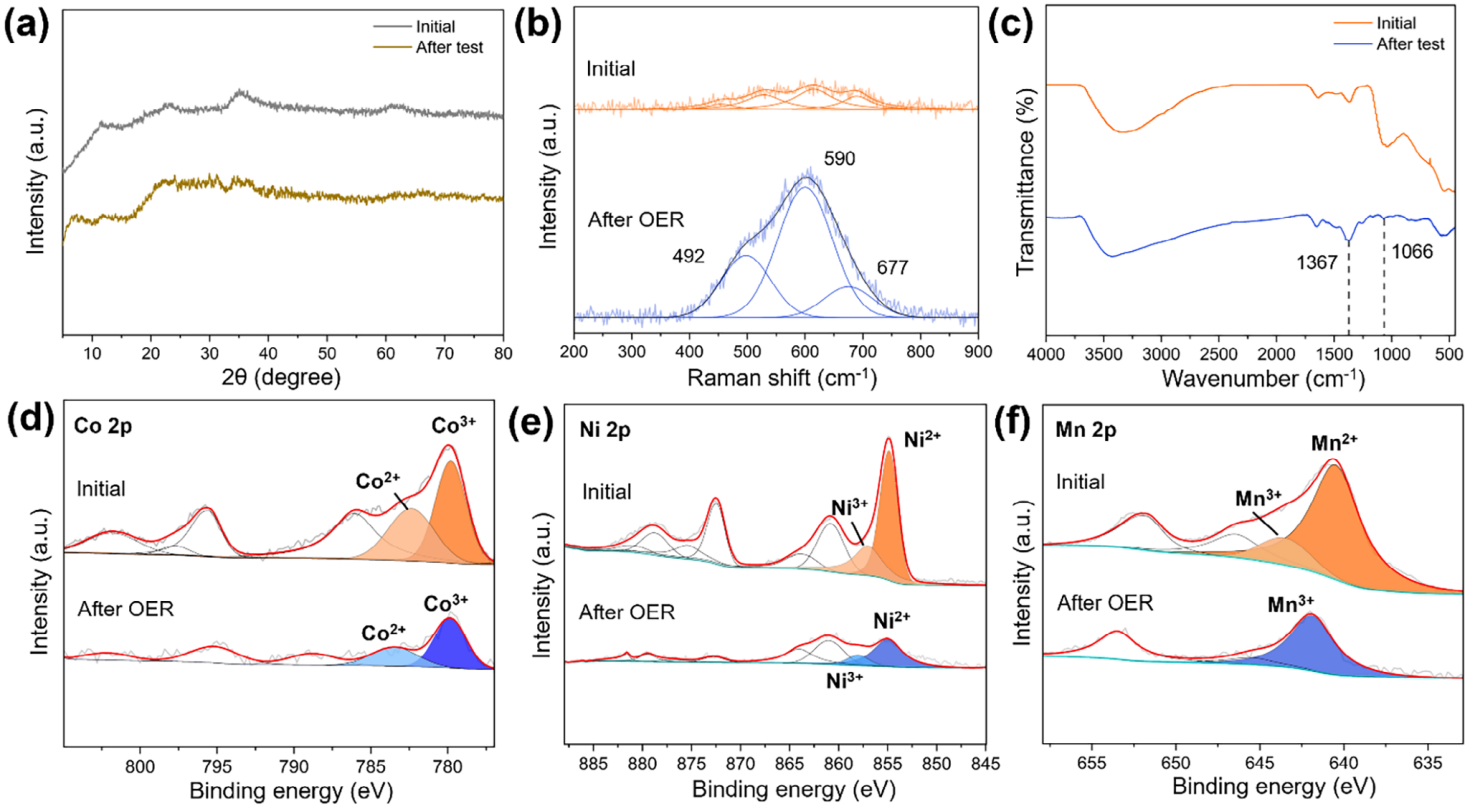
Structure and chemical constituent characterization of Q‐LDH‐0.1 after the durability test. a) XRD patterns. b) Raman spectra. c) FT‐IR spectra. XPS spectra of d) Co 2p. e) Ni 2p. f) Mn 2p.

To get better recognition of the structural stability of Q‐LDH‐0.1 after OER test, we performed a series of characterizations on Q‐LDH‐0.1 after the 80 h chronoamperometry test. After OER process, the color of Q‐LDH‐0.1 shifted from brown to black (Figure S12, Supporting Information), likely attributed to the oxidation of divalent cations within the LDH. Despite this, the Q‐LDH‐0.1 preserved its original blocky morphology and uniform element distribution (Figures S13 and S14, Supporting Information). Though there are slight changes in the intensities of all peaks in the XRD pattern (**Figure** **6**a), the four distinct diffraction peaks remain the same, which suggests that the Q‐LDH‐0.1 retained its hydrotalcite crystal structure after the OER process. The Raman spectra of Q‐LDH‐0.1 after OER test show three peaks with significantly higher intensities compared to the initial state (Figure 6b). The peak at 492 cm^−1^ is assigned to NiOOH,^[^ ^31^, ^45^ ^]^ which can facilitate the OER process by accelerating the formation of intermediate species (OOH*).^[^ ^46^ ^]^ The peak at 590  and 677 cm^−1^ are ascribed to stretching modes of γ‐CoOOH^[^ ^47^ ^]^ and Co oxides,^[^ ^48^, ^49^ ^]^ respectively. The peaks at 3340  and 1636 cm^−1^ in FT‐IR spectra remain unchanged (Figure 6c). The peak at 1066 cm^−1^ significantly diminishes, which can be attributed to more OH^−^ and CO_3_^2−^ being inserted into hydrotalcite layers during the OER process, thereby decreasing the amount of PO_4_^3−^ in the interlayer space. Meanwhile, the peak at 1367 cm^−1^, corresponding to CO_3_^2−^, increases slightly. The chemical states of metal elements were investigated by XPS. The survey spectrum further confirms the existence of Fe, Ni, Co, and Mn after the test (Figure S15, Supporting Information). As revealed by Figure 6d, a large part of Co was oxidized to Co^3+^ after the durability test, and similarly, a large portion of Ni^2+^ and almost all of Mn^2+^ were oxidized to their corresponding trivalent cations (Figure 6e,f). The iron predominantly remains as Fe^3+^, however, its characteristic peak exhibits a slight shift toward lower binding energies, indicating subtle changes in the electronic environment (Figure S16, Supporting Information). Numerous studies have demonstrated that high‐valence state metal sites, especially Ni, Co and Mn, are crucial for enabling OER by strengthening the adsorption of hydroxyl ions, facilitating charge transfer, and accelerating the transformation of intermediate species.^[^ ^50^, ^51^, ^52^, ^53^, ^54^ ^]^ Here, it can be concluded that under the optimal iron addition, Q‐LDH‐0.1 is most prone to forming several active metal species during the OER process, which, combined with the synergistic effects among the metal cations and the involvement of LOM, leads to an overall enhancement in OER performance.

## 3 Conclusion

In summary, we have successfully synthesized a series of quaternary FeCoNiMn hydrotalcites by reusing NCM and LFP cathode materials as precursors, achieving remarkable catalytic activity for OER. The synthesis process involves thermal leaching, followed by co‐precipitation and hydrothermal procedure to obtain the final LDHs. All the metal cations in the LDHs show mixed valence state between bivalence and trivalence, which combined to produce a highly disordered mixture with uniform distribution of all the metal cations. The performance analysis suggests that during the OER process, the incorporation of Fe cations in Q‐LDH‐0.1 introduces additional active sites and strengthens strong electronic interactions with other cations. These interactions modify the OER pathway, promoting the involvement of the LOM and ultimately enhancing the catalytic performance. These combined effects result in a minimal overpotential of 270 mV to reach a current density of 10 mA cm^−2^, demonstrating superior catalytic activity compared to all other catalysts samples in this study and exhibiting competitive performance relative to previously reported catalysts. This research provides a feasible pathway for recycling end‐of‐life lithium battery cathodes and offers valuable scientific insight into OER electrocatalyst development.

## 4 Experimental Section

### Raw Materials

NCM cathodes black powder was purchased from Xinxiang Zhuoyuan renewable resources Co., LTD. H_2_SO_4_ (AR), HCl (AR), NaOH (AR) and tetramethylammonium hydroxide (TMAOH) were purchased from Shanghai Aladdin Bio‐Chem Technology Co., LTD. The Nafion solution (5 wt.%) and conductive carbon (XC‐72R) were purchased from Yueci (Shanghai) Electronics Technology Co., Ltd. The nickel foam (thickness: 0.2 mm) was obtained from Eryi (Wuhu) Materials Technology Co., Ltd, and was ultrasonically cleaned for 20 min using isopropanol, diluted hydrochloric acid, and deionized water, respectively, before use. All other chemicals were used without further purification, deionized water (18.2 MΩ) was used throughout the experiment.

### Material Synthesis

100 g NCM powder was added into 500 mL of 4 m HCl aqueous solution, the mixture was stirred to dissolve and heated in an oil bath at 90 °C for 5 h, then purified by filter paper to remove the precipitate. The obtained filtrate was designated as acid leachate. 10 mL of acid leachate was applied every time as precursor, while different amounts of FePO_4_ were added in it to achieve Fe ion concentrations of 0.01 , 0.05 , 0.1 , 0.2 , 0.3 m, and appropriate amount of concentrated sulfuric acid was added at the same time to ensure complete dissolution of FePO_4_. After that, the pH of the solution was adjusted to 10 with 1 m NaOH solution for precipitation, and the obtained precipitate was placed in a 100 mL stainless steel autoclave and reacted at 120 °C in a blast drying oven for 8 h. After being centrifuged and washed several times with deionized water, the precipitation was purified, which was later dried at blast drying oven for at least 12 h and applied as quaternary layered double hydroxides (Q‐LDHs) catalysts. They are named as Q‐LDH‐0.01, Q‐LDH‐0.05, Q‐LDH‐0.1, Q‐LDH‐0.2, and Q‐LDH‐0.3, respectively, according to the diverse content of Fe.

For comparison, the Ternary‐LDH was prepared using the same method but without the addition of FePO_4_. The obtained sample was denoted as T‐LDH.

### Material Characterization

The crystal structure was detected via XRD (X'Pert3 Powder) at room temperature, with Cu Kα radiation of  λ = 1.54178 Å operating at 40 kV, 200 mA, scan rate of 5° min^−1^ and range of 5° to 80°. The morphology and elemental distribution of the samples were characterized by SEM (SU8010) and HRTEM (JEM‐2100F). A detailed elemental composition analysis was obtained through ICP‐OES (5800). BET (Micromeritics ASAP 2460) was conducted to obtain the surface area and pore‐size information. FT‐IR spectroscopy (Spectrum 400F) was performed to investigate the molecular composition and chemical bonds within the range of 400–4000 cm⁻¹. Raman spectroscopy (LabRAM HR) with a 532 nm excitation wavelength was used to analyze the chemical structure and molecular interactions of the samples, in situ Raman test was conducted during chronoamperometric measurements in 1 M KOH. The elemental composition and chemical valence states were further investigated using XPS (ESCALAB250).

### Electrochemical Measurement

All the electrochemical measurements were conducted using a standard three‐electrode cell on CHI660E (CHI, Shanghai) electrochemical workstation. The catalysts ink of Q‐LDH or T‐LDH was prepared by mixing 2 mg of active material and 0.5 mg of conductive carbon (XC‐72R) with 490 µL of deionized water and ethanol (volumetric ratio of 1:1), and 10 µL of 5 wt.% Nafion solution was added as binder, then ultrasonically mixed for 30 min. 20 µL of obtained catalyst ink was loaded on a glassy carbon electrode as the working electrode with loading amount of 0.4 mg cm^−2^. Graphite rod was applied as the counter electrode and Hg/HgO electrode as the reference electrode. The electrolyte is 1 m KOH aqueous solution (pH ≈14). Prior to each test, oxygen is bubbled into the electrolyte for at least 40 min using an aeration tube to ensure the solution is oxygen‐saturated, thus maintaining the H_2_O/O_2_ equilibrium potential at 1.23 V (vs Reversible Hydrogen Electrode, RHE).

CV scans were performed to activate the working electrode before the electrochemical oxygen evolution reaction test, with a scanning voltage range of 1.2–1.8 V (vs RHE) and scan rate of 50 mV s^−1^ for 20 cycles. LSV was conducted in the voltage range of 1.2–1.8 V (vs RHE) and a scan rate of 10 mV s^−1^, applying 95% auto iR compensation. C_dl_ was determined by performing CV tests at different sweep rates within a non‐Faradaic potential window (in this study it's 0.1–0.2 V (vs Hg/HgO)), at diverse scan rates of 20, 40, 60, 80, 100, and 120 mV s^−1^. Plot the half current density difference at 0.15 V (j = 1/2 (j_a_ – j_c_)) against the scan rate, and the slope gives the C_dl_. EIS analysis was performed at the open‐circuit potential of each catalyst with a sinusoidal amplitude of 5 mV in the frequency range from 10^−1^  to 10^5^ Hz. Electrochemical stability was detected via chronoamperometry on a nickel foam (with the same catalysts loading amount of 0.4 mg cm^−2^) at a constant voltage of 1.51 V (vs RHE) over a duration of 80 h.

### DFT Calculation

Density functional theory (DFT) calculation was conducted using the Vienna Ab initio Simulation Package (VASP)^[^ ^55^, ^56^ ^]^ with the PBE‐GGA exchange‐correlation functional for projector augmented wave (PAW) pseudopotentials.^[^ ^57^, ^58^ ^]^ An energy cutoff of 380 eV was employed, along with a 4×6×2 Monkhorst‐Pack k‐point grid. During structural optimizations, the convergence criterion for Hellmann–Feynman forces was set to 1.5×10^−2^ eV Å^−1^, while the energy convergence for the electronic wave function was set to 10^−4^ eV. To account for dispersion interactions, van der Waals interactions were included using the DFT+D3 correction method.^[^ ^59^ ^]^ Additionally, Hubbard U corrections were applied with values of 6.2, 3.9, 5.3, and 3.3 eV for Ni, Mn, Fe, and Co, respectively, to accurately capture the localized electronic states of the metallic atoms in the composite electrode.^[^ ^60^, ^61^ ^]^ The T‐LDH and Q‐LDHs structures were composed of NiFe_x_MnCo (x = 3%, 6%, 13%, 22%, 34%, and 44%, respectively) with interlayer anions OH^−^, CO_3_^2−^, and Cl^−^.

## Conflict of Interest

The authors declare no conflict of interest.

## Supporting information



Supporting Information

## Data Availability

The data that support the findings of this study are available from the corresponding author upon reasonable request.
