# Supplementary material for: Recycling Defunct Lithium‐Ion Battery Cathodes to Quaternary Layered Double Hydroxides for Efficient Oxygen Evolution Reaction
Source: Adv Sci (Weinh). 2025 Apr 8;12(26):2501957. doi: 10.1002/advs.202501957 (PMC12245122; doi:10.1002/advs.202501957)
Supplement: Supplementary file 1 — Supporting Information [file ADVS-12-2501957-s001.docx]

Supporting Information

Recycling Defunct Lithium-Ion Battery Cathodes to Quaternary Layered Double Hydroxides for Efficient Oxygen Evolution Reaction

Ronghou Yao, Jin Wu, Shivam Kansara, Zhaowei Sun*, Hyokyeong Kang, Feng Liu, Kaizhao Wang, Jin Hu, Xiangming Li*, Dapeng Wu*, Jang-Yeon Hwang, Shizhao Xiong*

Ronghou Yao, Prof. D Wu

School of Environment, Henan Normal University, Xinxiang, Henan 453007, China.

Ronghou Yao, Jin Wu, Prof. S. Xiong, Dr. K. Wang, Dr. Z. Sun, Prof. X Li, Prof. Jin Hu

Faculty of Material Science and Engineering, Kunming University of Science and Technology, Kunming, 650093, China.

Dr. S. Kansara, Prof. J-Y Hwang, Hyokyeong Kang

Department of Energy Engineering, Hanyang University, Seoul, S Korea.

Dr. Feng Liu

Qujing No.1 Hospital, Affiliated Qujing Hospital of Kunming Medical University, No.1 Yuanlin Road, Qujing City, Yunnan Province, China.

E-mail address: 20230231@kust.edu.cn (Z. Sun), lixm@kust.edu.cn (X. Li), dapengwu@htu.edu.cn (D. Wu), shizhao.xiong@kust.edu.cn (S. Xiong).

**Table S1.** Metal elemental concentration analysis results for synthesized catalysts by ICP-OES test, the proportions displayed in Figure 2c were calculated based on the results.

| Content | Co (μg L^-1^) | Fe (μg L^-1^) | Mn (μg L^-1^) | Ni (μg L^-1^) |
| --- | --- | --- | --- | --- |
| T-LDH | 0.08191 | 0.01201 | 0.10142 | 0.17162 |
| Q-LDH-0.01 | 0.06055 | 0.01688 | 0.07614 | 0.12081 |
| Q-LDH-0.05 | 0.08299 | 0.05573 | 0.11366 | 0.17343 |
| Q-LDH-0.1 | 0.05870 | 0.06821 | 0.07130 | 0.11562 |
| Q-LDH-0.2 | 0.04997 | 0.11240 | 0.06880 | 0.10112 |
| Q-LDH-0.3 | 0.04965 | 0.16944 | 0.07008 | 0.10006 |


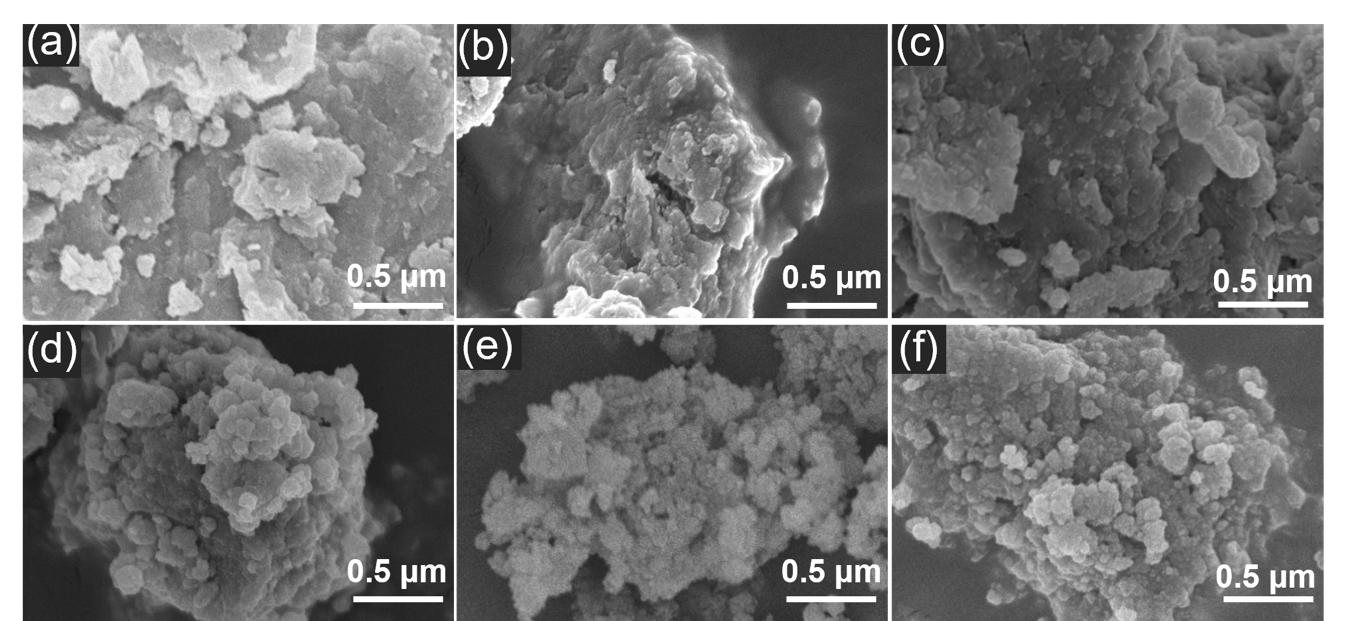


**Figure S1.** SEM images of T-LDH and Q-LDHs powders. (a) T-LDH. (b) Q-LDH-0.01. (c) Q-LDH-0.05. (d) Q-LDH-0.1. (e) Q-LDH-0.2. (f) Q-LDH-0.3.


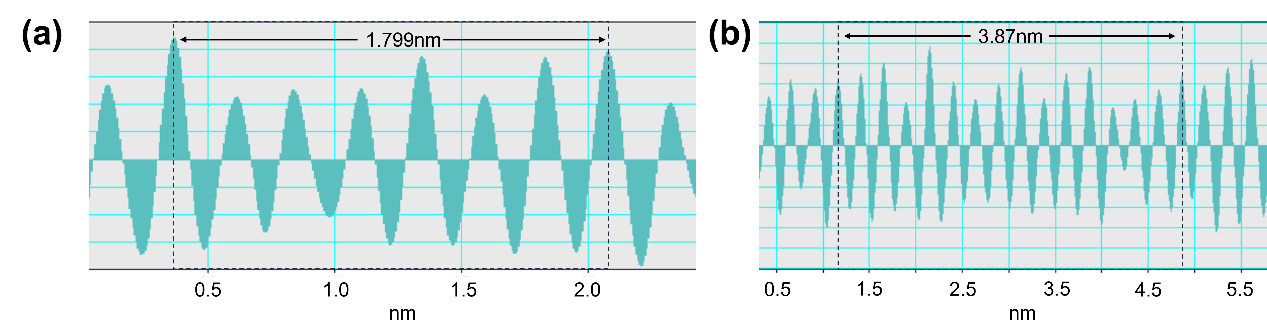


**Figure S2.** Distribution diagram of lattice fringe obtained by Digital-Micrograph of (a) T-LDH. (b) Q-LDH-0.1.


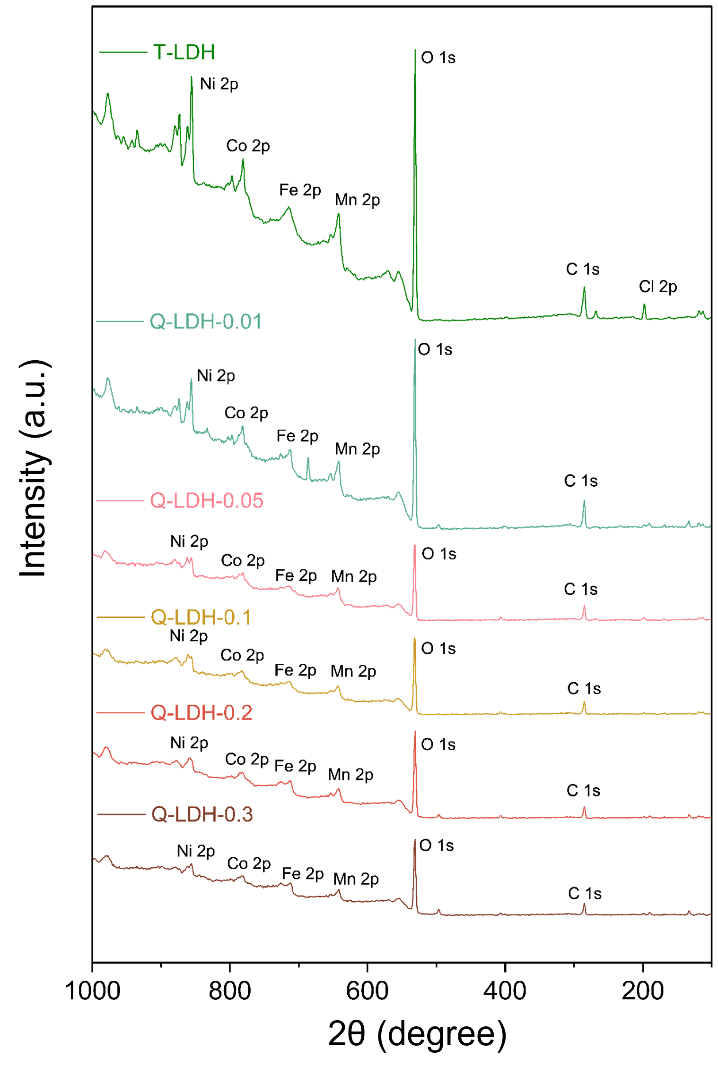


**Figure S3.** Normalized XPS survey spectra of T-LDH and Q-LDHs.


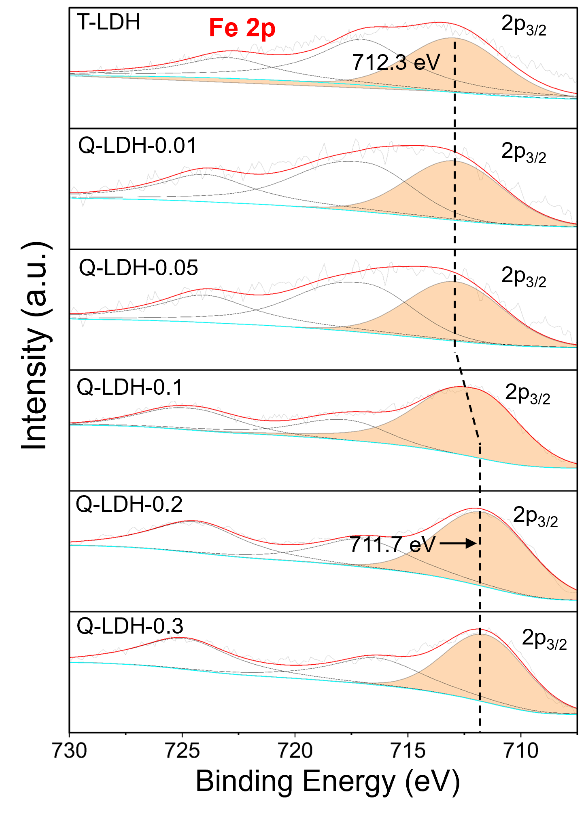


**Figure S4.** Fe 2p XPS spectra of T-LDH and Q-LDHs.


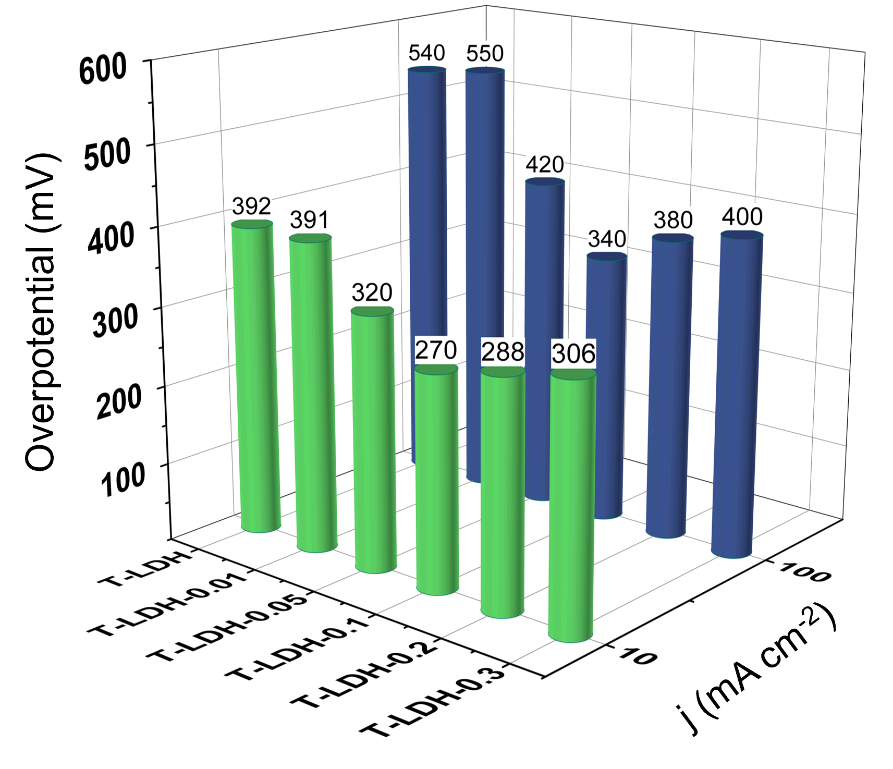


**Figure S5.** OER overpotentials at given current densities of 10 mA cm^-2^ and 100 mA cm^-2^ for T-LDH and Q-LDHs.


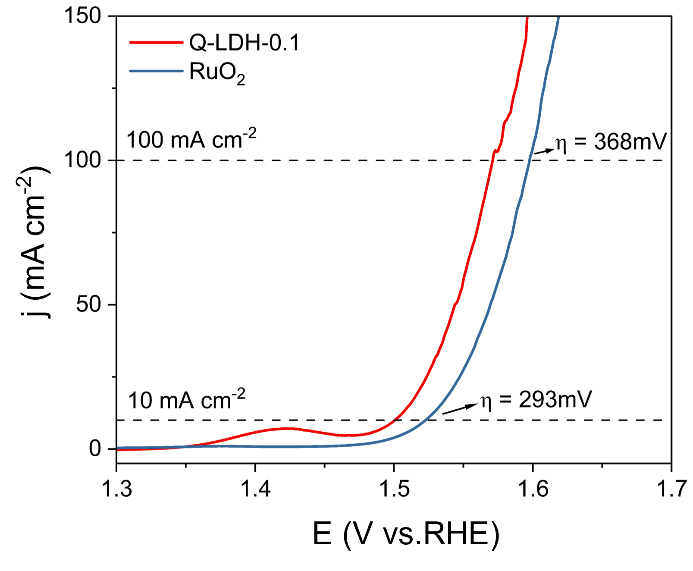


**Figure S6.** LSV curves (the scan rate is 10 mV s^−1^) with 95% iR correction of Q-LDH-0.1 and RuO_2_. The test was conducted with standard three-electrode system in 1M KOH electrolyte, with a graphite rod as the counter electrode and a Hg/HgO electrode as the reference electrode.

**Table S2.** The electrochemical testing condition of recycled cathode-derived OER catalysts reported in other studies that cited in Figure 4b.

| Catalysts name | Electrolyte | Scan rate (mV s^-1^) | Working electrode substrates | Mass loading (mg cm^-2^) |
| --- | --- | --- | --- | --- |
| Q-LDH-0.1 (Our work) | 1 M KOH | 10 | Glassy carbon | 0.4 |
| Ni-LiFePO_4_ | 1 M KOH | 5 | Carbon paper/glassy carbon | 0.42 |
| LNCM-G/CC | 1 M KOH | 5 | In-situ grown on carbon cloth | —— |
| Mn_2_O_4_ | 1 M KOH | 5 | Carbon cloth | 0.105 |
| CP3 | 1 M KOH | 10 | Carbon cloth | —— |
| s-Co_3_O_4_ | 1 M KOH | 2 | Glassy carbon | —— |
| NCM-OH | 1 M KOH | 5 | Carbon cloth | 0.53 |
| NiCoMnFe-LDH/C-50 | 1 M KOH | 5 | Glassy carbon | 1 |
| 6.8-Co/NFCH-FF | 1 M KOH | 5 | In-situ grown on Fe foam | —— |


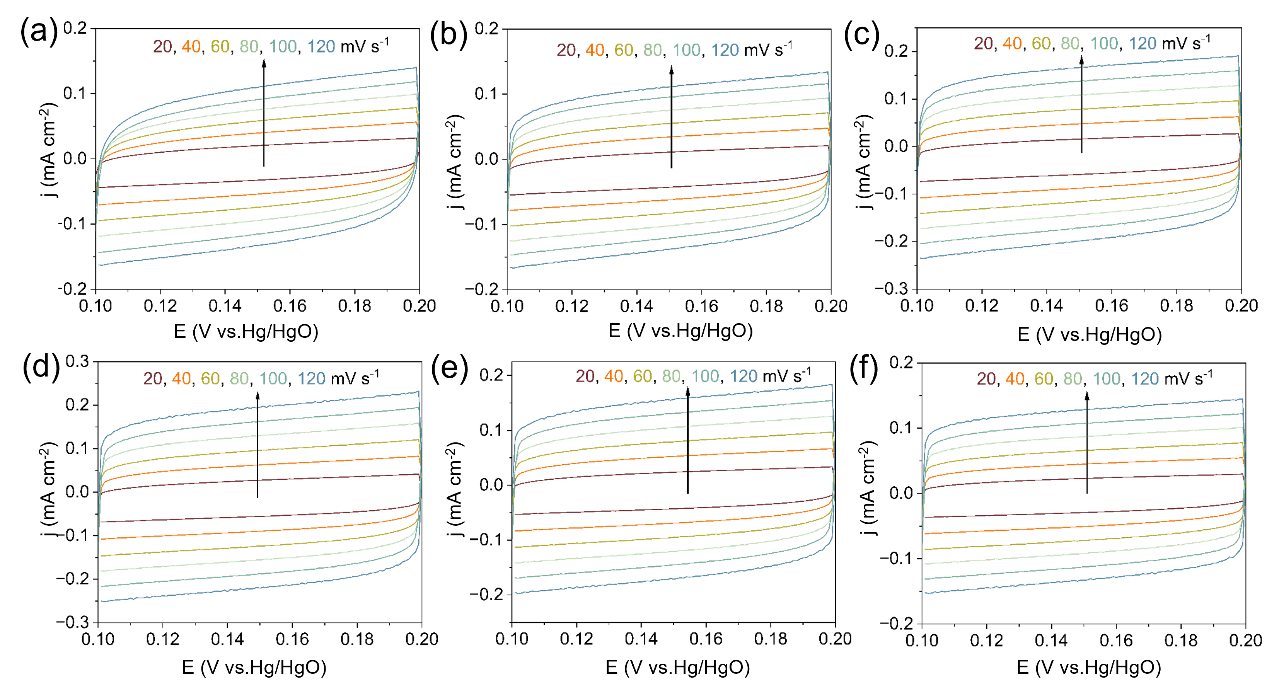


**Figure S7.** Cyclic voltammetry curves at different scan rates for electrochemical surface area (ECSA) tests in 1 M KOH. (a) T-LDH. (b) Q-LDH-0.01. (c) Q-LDH-0.05. (d) Q-LDH-0.1. (e) Q-LDH-0.2. (f) Q-LDH-0.3.


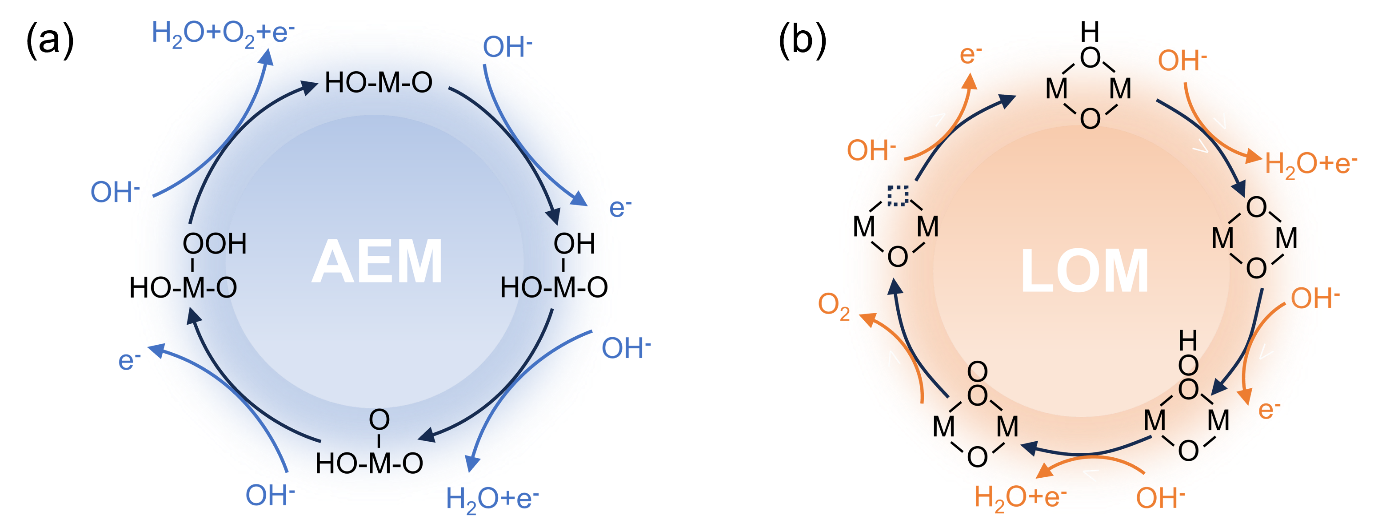


**Figure S8.** Schematic illustrations of the (a) AEM and (b) LOM pathways for OER.


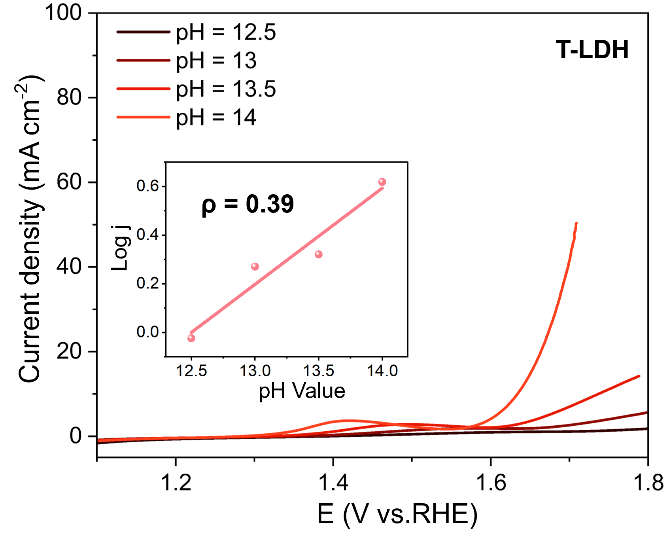


**Figure S9.** LSV curves of T-LDH measured at different pH values (12.5-14).


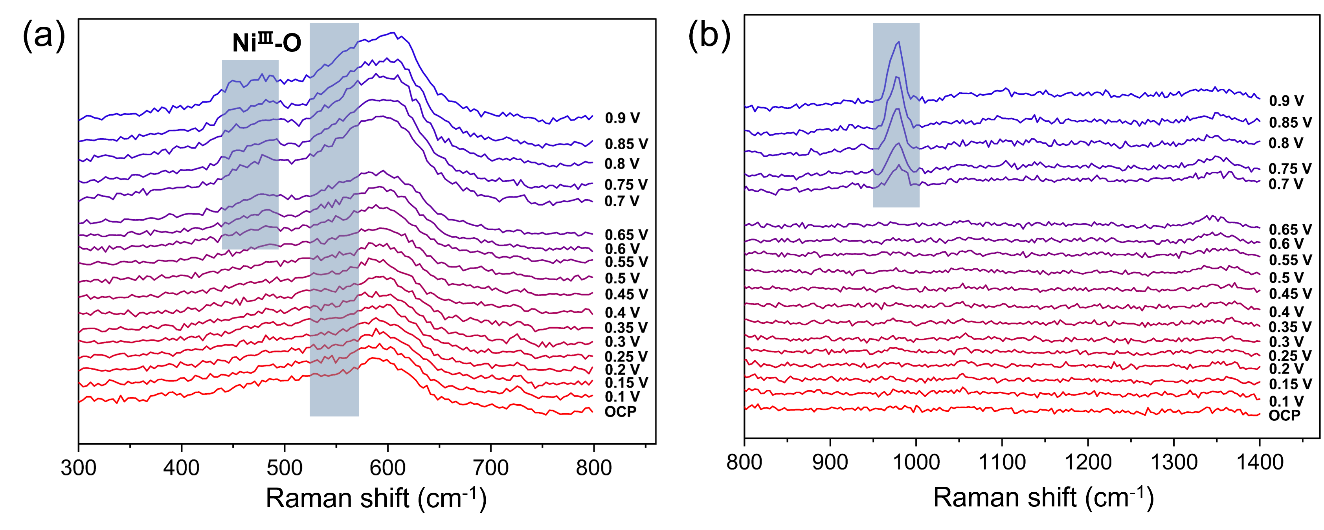


**Figure S10.** In-situ Raman spectra on Q-LDH-0.1 in the (a) 300-800 cm^-1^ and (b) 800-1400 cm^-1^.


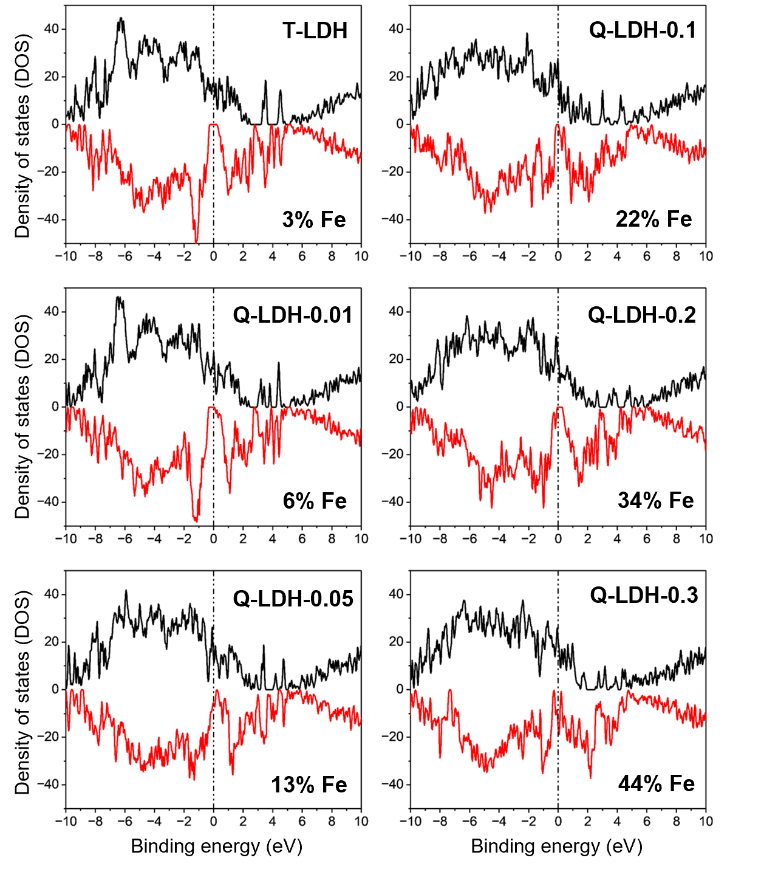


**Figure S11.** DOS plots of T-LDH and Q-LDHs.


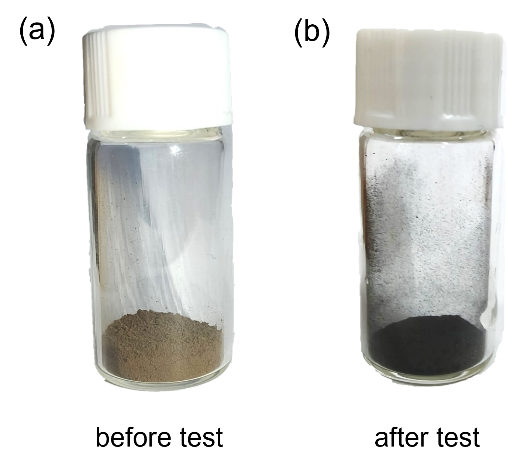


**Figure S12.** Optical photos of Q-LDH-0.1 sample a) before and b) after the durability test.


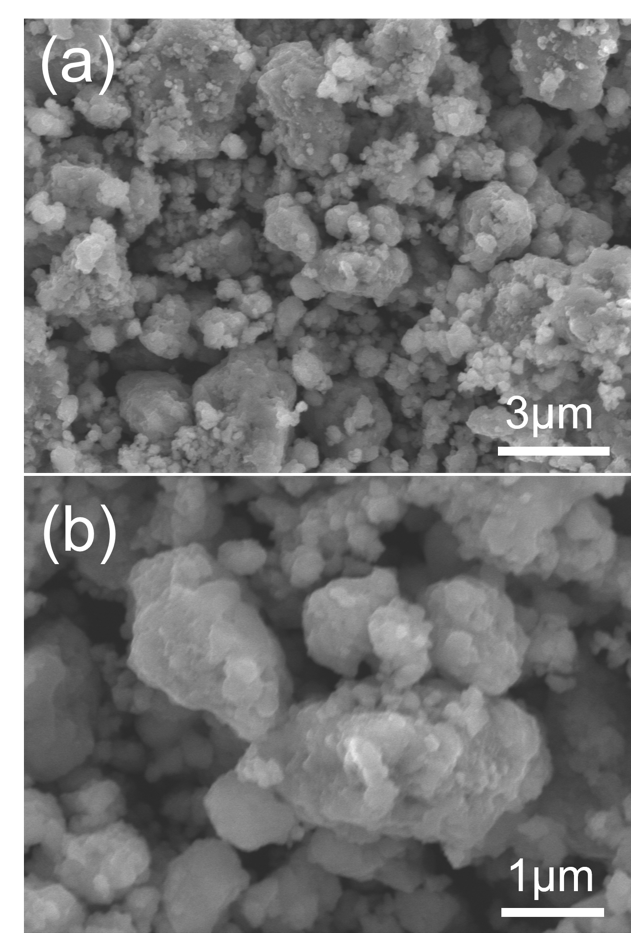


**Figure S13.** SEM images of Q-LDH-0.1 after the 80 h durability test.


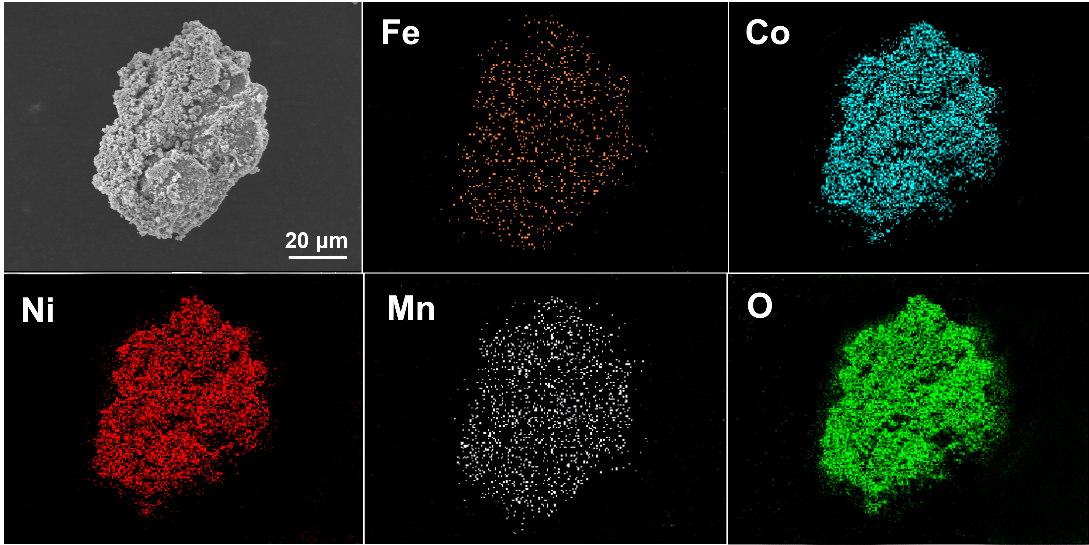


**Figure S14.** EDS mapping images of Q-LDH-0.1 after the 80 h durability test.


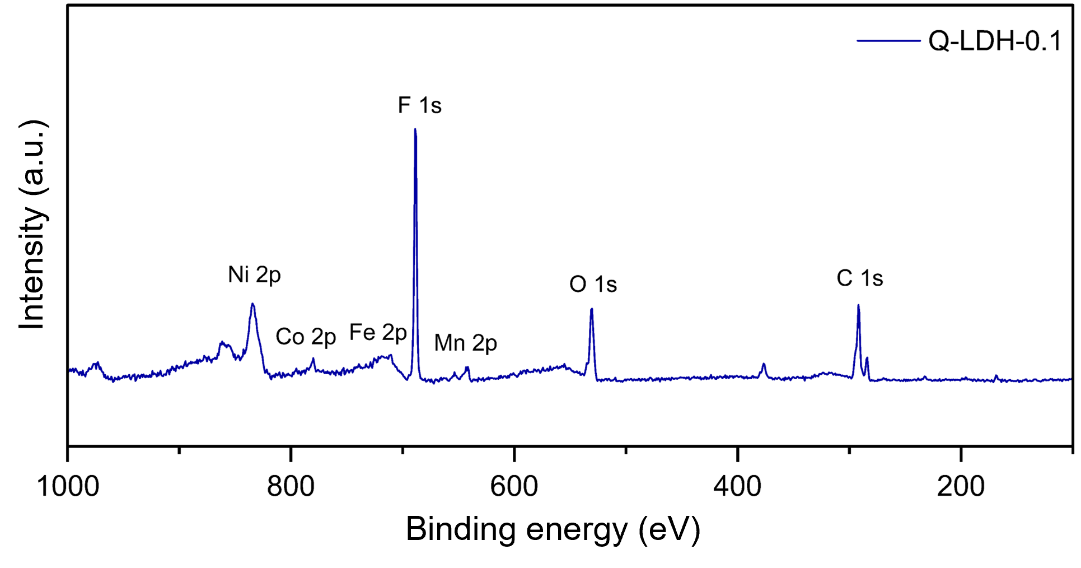


**Figure S15.** XPS survey spectrum of Q-LDH-0.1 after the durability test. The observed fluorine peak is attributed to the Nafion added during the ink preparation process.


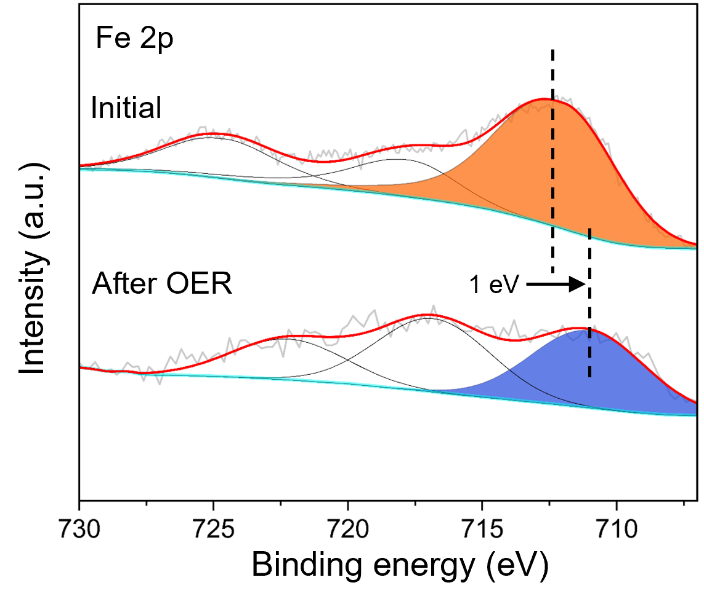


**Figure S16.** XPS spectra of Fe 2p of Q-LDH-0.1 before and after the durability test.
